# Interpersonal dyadic influences on transitions between pain states: a narrative review and synthesis

**DOI:** 10.1097/j.pain.0000000000003544

**Published:** 2025-02-21

**Authors:** Hollie Birkinshaw, Amanda C. de C Williams, Claire Friedrich, Charlotte Lee, Edmund Keogh, Christopher Eccleston, Tamar Pincus

**Affiliations:** a School of Psychology, University of Southampton, Southampton, United Kingdom; b School of Primary Care, Population Sciences, and Medical Education, University of Southampton, Southampton, United Kingdom; c Research Department of Clinical, Educational and Health Psychology, University College London, London, United Kingdom; d Department of Psychiatry, University of Oxford, Oxford, United Kingdom; e Centre for Pain Research, The University of Bath, Bath, United Kingdom; f Department of Experimental, Clinical and Health Psychology, Ghent University, Ghent, Belgium; g Department of Psychology, The University of Helsinki, Helsinki, Finland; h Faculty of Environmental and Life Sciences, University of Southampton, United Kingdom

**Keywords:** Pain, Transitions, Chronic pain, Psychosocial, Interpersonal, Dyadic, Parents, Partners

## Abstract

Pain is not experienced in isolation; it is affected by and affects other people. Interactions between parents and partners and people living with pain affect beliefs, emotions and behaviours, and pain progress and change. We searched systematically for longitudinal studies of associations between specific familial, dyadic, interpersonal factors and quantitative pain transitions. We coded studies for risk of bias. For the narrative synthesis, we grouped findings by dyads—parents and children, and people with pain and their partners (usually spouses), and then by the psychosocial mechanism/s. We described certainty of evidence for each pain transition and each mechanism. Patient and public contributors were involved throughout. Of 52 studies, 38 were of parents and children (27,814 dyads) and 14 of partners (4904 dyads). Three groups of predictive factors were identified for parent and child studies: parent mental health, parent cognitions, and parent behaviours. Parental anxiety (but not depression) predicted children's onset of pain and worsening; the evidence was of moderate certainty and almost exclusively involved mothers. Evidence that some parental behaviours, such as protective behaviours, were associated with worse child pain was of very low certainty. The evidence for partners was of poor quality, precluding synthesis. The review highlights that most interpersonal pain research fails to capture the complex dynamics of longstanding relationships and highlights the difficulty of doing so using simple models.

## Abstract 2

Supplemental Digital Content is Available in the Text.

## 1. Introduction

Pain is a ubiquitous human experience that changes over time. Pain transitions describe changes in pain states in terms of chronicity and severity. Recently, we established a framework outlining 5 pain states defined by chronicity (acute or chronic) and impact (low or high).^18^ From these states, 10 transitions were proposed, broadly categorised into onset of chronic pain (acute to chronic), change in chronic pain (worsening, improving, resolving), and maintenance (no change). Determining how and why people transition between these pain states is an important challenge and cannot be predicted from clinical, demographic, or biological factors alone; psychosocial mechanisms are also critical to understanding these changes.^20,38,62^ Although such mechanisms can include individual psychological factors, the wider set of social and contextual factors must also considered.^29^ Particularly important is pain behaviour occurring in social settings. Chronic pain is not experienced in social isolation; influences from significant others, family, and social networks on pain are cyclical and iterative, leading to a dynamic pain experience. Pain behaviour is often relational, signalling the need for care, and suppressed in social situations that represent possible threat.^3,17^ Therefore, it is also critical to capture the longitudinal impact of interpersonal factors on pain transitions.

A number of models represent interactions between family members, including the Interpersonal Fear Avoidance Model for parents and children, operant learning, communal coping, and intimacy for partners.^8,22^ Yet longitudinal research in this area is lacking. A comprehensive review and meta-analysis of the impact of parental cognitive, behavioural, and affective factors on child pain highlighted the impact on the child's outcomes of parental catastrophizing, protective behaviours, anxiety, depression, and stress.^15^ However, 50 of the 54 included studies were cross-sectional, and therefore could not be interpreted causally. The effects of partner/patient interactions on pain outcomes and transitions are also unknown; most reviews focus on couple-based interventions for chronic pain management,^40,71^ showing small but significant effects on pain and disability. A review of couples' interactions on chronic pain coping, for example, found mixed results for negative partner responses, and preliminary evidence for partner validation and invalidation.^57^ However, the majority of studies in this review were cross-sectional, limiting applicability to pain transitions.

In the absence of a synthesis of studies with clear timelines, the current evidence cannot identify possible causal relationships between interpersonal factors and pain transitions, and whether any mechanisms are shared across different dyadic relationships. This is critical, as the study of these interactions and their impact on pain transitions could identify unrecognised mechanisms. Furthermore, if any of these processes are modifiable, there is potential to develop new interventions to prevent the development of chronic pain, and to mitigate chronic pain. Therefore, this study aimed to conduct a synthesis of studies with at least 2 assessment points to collate directional relationships supported by evidence, and their relationship to theoretical models. This ultimately will enable an understanding of priorities for future research.

## 2. Methods

### 2.1. Protocol and registration

The protocol for this review was registered on Open Science Framework on 28th April 2022 and is available at: https://osf.io/q8gan/. This study was part of a larger set of reviews being undertaken by the CRIISP (Consortium to Research Individual, Interpersonal, and Social influences in Pain; https://criisp.uk/), where a shared approach was adapted.

### 2.2. Eligibility criteria

#### 2.2.1. Study design

We included empirical studies investigating a familial, dyadic, interpersonal factor that had a quantitative pain outcome and at least 2 assessment points, including prospective cohort studies, experimental studies, and randomised controlled trials. Studies were required to be completed and peer-reviewed. We excluded cross-sectional studies, qualitative studies, protocols, case studies, dissertations, book chapters, and studies involving animals.

We ran 2 searches to represent the 2 types of dyads included:

(1) Parents or caregivers, and children (including babies, children, and young adults).
(2) People with pain, and their partners (including spouses and significant others).

To be eligible, studies were required to test factors related to interactions between a) children with pain and a parent or carer, and b) adults with pain and a significant other, typically their partner. There were no restrictions related to pain duration or pain type.

#### 2.2.2. Participants

Participant eligibility varied for each search. For the parent and child search, we included people of any age. For the partner search, this was restricted to adults only. There were no restrictions on any other participant characteristics. We included pain of any type and duration.

#### 2.2.3. Outcomes

In line with the aim to assess pain transitions, studies were required to have a pain-related outcome to be included. Examples of pain related outcomes included:

(1) Pain intensity (eg, measured on a 0-10 numerical rating scale).
(2) Pain interference (eg, as measured by the Brief Pain Inventory).
(3) Presence of pain (eg, the presence of chronic pain 6 months after surgery).
(4) Pain-related functioning (eg, Functional Disability Inventory).

Where possible, we also explored transitions between pain states.^18^ These were classified as:

(1) Onset of pain: the development of either acute or chronic pain between observations
(2) Worsening: the intensity and/or impact of pain increasing between observations (eg, worse pain intensity or disability scores)
(3) Improving: the intensity and/or impact of pain decreasing between observations (eg, better pain intensity or disability scores)
(4) Maintenance: no change in the intensity and/or impact of pain between observations
(5) Resolving: no pain reported at the second observation

### 2.3. Sources and searches

Four databases (Embase, MEDLINE, PsycINFO, and Web of Science) were searched from inception until 28th April 2024. A backward and forward search of included records was conducted, and reference lists of relevant published reviews were hand-searched. The search strategies are available in the supplemental digital content (http://links.lww.com/PAIN/C225). Studies were restricted to those published in English.

### 2.4. Screening and selection of studies

Studies were screened using Rayyan.^50^ Titles and abstracts were single screened by 2 reviewers (C.F. and H.B.), split 50/50%, and 10% of records were randomly selected and screened by both reviewers. If there was more than 10% disagreement, a further 10% of abstracts were screened until there was less than 10% disagreement. Any conflicts were resolved via discussion between C.F. and H.B.; if a decision could not be made, then a third author arbitrated (T.P.).

Full texts of eligible studies were then sought. Where full texts were unavailable to be retrieved, publication authors were contacted and asked to share. All full texts were independently screened by 2 reviewers (C.F. and T.P.). Any discrepancies were discussed and resolved, and a third reviewer (H.B.) was consulted if necessary.

### 2.5. Data extraction

Data regarding study information and methods, participant demographics, findings, and pain transitions were extracted for each study. Data extraction was done by either C.F. or H.B., with 10% of each type of study (eg, 10% of randomised controlled trials, 10% of cohort studies) checked by the other author. Very few errors were identified when checked. Data extraction was undertaken using a data extraction form that was agreed across workpackages within the CRIISP consortium. The data extraction form was piloted with 2 studies from each workpackage and amended accordingly before full data extraction began.

### 2.6. Risk-of-bias assessment

We assessed each study's risk of bias using tools appropriate to the study methodology.

(1) For cohort studies, we used the QUIPs tool.^23,24^
(2) For randomised controlled trials, we used the Cochrane Risk of Bias Tool 1.0.^27^
(3) For experimental studies, we used the Joanna Briggs Checklist for Quasi-Experimental Studies.^79^

Risk-of-bias assessment was undertaken by either C.F. or H.B., with 10% of each study type checked by the other author. Disagreements were resolved through discussion; if no resolution could be found, then a third author (T.P.) arbitrated. Overall ratings of “high”, “moderate”, and “low” risk of bias were given to each study.

### 2.7. Data synthesis

There was substantial heterogeneity across included studies in design, duration, measures, and statistical analyses, which precluded quantitative analysis. Therefore, we conducted a narrative synthesis, grouping our findings firstly by dyads, and secondly by the psychosocial mechanisms identified. Findings across studies are summarised, and the evidence for pain transitions outlined. To ensure a measure of reliability, only mechanisms investigated by 2 or more studies are included in the narrative synthesis. Results from all studies for each mechanism are reported in the Summary of Findings tables.

### 2.8. Assessment of certainty of the evidence

We assessed the certainty of the evidence for each pain transition identified for each mechanism using the GRADE approach. As this was a narrative review, we were unable to explore imprecision or publication bias because of a lack of a pooled estimate. Therefore, studies were downgraded for the following reasons:

(1) Risk of bias—concerns about limitations in the study design and execution of individual studies leading to overestimation or underestimation of event rates (eg, follow-up duration, lack of representative and well-defined samples, no adjustment for important prognostic factors, etc). These judgments were made using the tools appropriate to each methodology; the proportions of studies with high risk of bias/severe concerns were added to guide GRADE judgments.
(2) Inconsistency—concerns regarding the inconsistency in risk estimates that was not explained by subgroup analyses.
(3) Indirectness—concerns regarding the population and outcomes within included studies were considered important to the research question.

GRADE assessment was undertaken by 2 authors (H.B. and C.L.).

### 2.9. Patient and public involvement and engagement

Patient and public involvement and engagement (PPIE) members from our CRIISP Workpackage Development Group (WDG) were involved in the review from inception. The WDG consists of 6 people living with chronic pain who have been involved in the study since the start. The WDG assisted in developing the search by suggesting potential mechanisms or populations that were missing; and were involved in the synthesis of the results, discussing the strength of evidence, what was missing, and what should be taken forward for further research.

## 3. Results

### 3.1. Study selection

**Figure 1.**
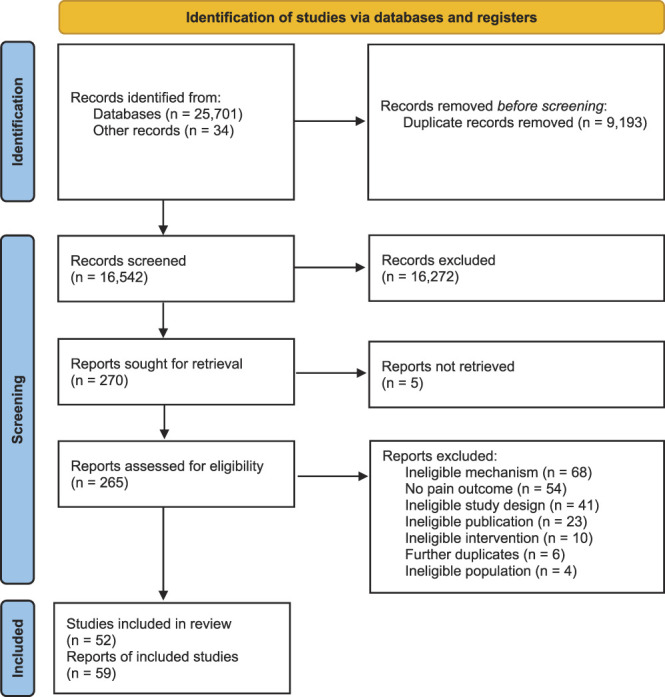
PRISMA diagram.

The search returned 25, 701 records through databases and 34 through other sources. After deduplication, we screened 16,826 records at title and abstract, and 270 records at full-text. Fifty-two studies were included (the PRISMA flowchart is presented in Fig. 1). One study^46^ reported results of 2 separate studies with different populations; therefore, these were analysed and reported as separate studies.

### 3.2. Included studies

We included 52 studies with a total of 32,718 dyads. The full study characteristics are available in Table 1.

**Table 1:** Study characteristics.

Study ID	Country	Study design	Dyads (n)	Setting	Pain type	Mechanisms	Risk of bias
Barnes 2017^1^	United States	Cohort	81	Inpatient hospital	Variety of chronic pain conditions	Parent mental health	High
Benore 2018^2^	United States	Cohort	670	Inpatient pain management	Variety of chronic pain conditions	Parent mental health	High
Beveridge 2021^6^	Canada	Cohort	192	Outpatient pain management	Variety of chronic pain conditions	Parent mental health	High
Beveridge 2024^5^	Canada	Cohort	76	Outpatient pain management	Variety of chronic pain conditions	Parent mental health Parent behaviours	Low
Birnie 2017^7^	Canada	Cohort	167	Inpatient hospital	Scoliosis	Parent cognitions	High
Carriere 2020^10^	United States	Cohort	124	Outpatient clinic	Knee osteoarthritis	Partner support	Low
Chow 2016^11^	United States	Cohort	195	Outpatient clinic	Variety of chronic pain conditions	Parent cognitions Parent behaviours	High
Connelly 2010^12^	United States	Cohort	9	Outpatient clinic	Juvenile idiopathic arthritis	Parent behaviours	High
Connelly 2017^13^	United States	Cohort	66	Outpatient clinic	Juvenile idiopathic arthritis	Parent behaviours	Low
Darlington 2012^15^	The Netherlands	Cohort	2230	General public cohort	Variety of chronic pain conditions	Parent mental health	High
Dougherty 2021^17^	United States	Cohort	155	Outpatient pain management	Amplified musculoskeletal pain (AMPS)	Parent cognitions Parent behaviours	High
Edlund 2015^20^	Sweden	Experimental	20	General public adverts	Any chronic pain	Partner validation	Low
Gere 2014^22^	United States	Cohort	152	Outpatient clinic	Knee osteoarthritis	Partner perceptions of patient self-efficacy	Moderate
Helgeland 2010^26^	Norway	Cohort	456	General public cohort	Abdominal pain	Parent mental health	High
Hemphill 2016^27^	United States	Cohort	152	Outpatient clinic	Knee osteoarthritis	Partner perceptions of patient self-efficacy	Low
Humberg 2024^29^	Germany	Cohort	689	General public cohort	Any chronic pain	Parent mental health Parent behaviours	Low
Kindt 2016^33^	Belgium	Cohort	70	Patient organisation	Any chronic pain	Partner helping motivations	High
Kindt 2020^32^	Belgium	Experimental	68	Patient organisation	Any chronic pain	Goal conflict	High
Koechlin 2022^34^	United States	Cohort	84	Outpatient clinic	Variety of chronic pain conditions	Parent cognitions Parent behaviours	High
Lalouni 2022^35^	Sweden	Cohort	90	Outpatient clinic	Functional abdominal pain	Parent cognitions Parent behaviours	High
Lam 2009^36^	Canada	Cohort	133	General public adverts	Rheumatoid arthritis	Partner depression	High
Law 2020^37^	United States	Cohort	239	Outpatient clinic	Migraine and tension-type headache	Parent cognitions Parent behaviours	Low
Li 2022^37^	Taiwan	Cohort	1139	General public cohort	Any chronic pain	Parent mental health	Moderate
Logan 2005^39^	United States	Cohort	78	Outpatient clinic	Recurrent abdominal pain or migraine headache	Parent mental health	High
Moore 2020^41^	United States	Cohort	50	General public cohort	General pain symptoms	Parent mental health	High
Mun 2021^42^	United States	Cohort	731	General public cohort	General pain symptoms	Parent mental health Parent behaviours	Low
Neville 2020^43^	Canada	Cohort	95	Outpatient pain management	Variety of chronic pain conditions	Parent mental health Parent cognitions Parent behaviours	Moderate
Neville 2021^44^	Canada	Cohort	152	Outpatient pain management	Variety of chronic pain conditions	Parent cognitions Parent behaviours	Moderate
Noel 2016^46^	Canada	Cohort	89	Outpatient clinic	Functional abdominal pain	Parent behaviours	High
Noel 2016^46^	Canada	Cohort	214	Outpatient pain management	Variety of idiopathic chronic pain conditions	Parent behaviours	High
O'Neill 2020^48^	United States	Cohort	162	Veterans database	General pain symptoms	Partner support	Moderate
Osterhaus 1997^49^	The Netherlands	RCT	39	General public adverts	Headache	Parent behaviours	High
Page 2013^51^	Canada	Cohort	83	Inpatient hospital	Postsurgical pain	Parent mental health Parent cognitions	High
Parker 2020^53^	United States	Cohort	113	Outpatient pain management	Variety of chronic pain conditions	Parent cognitions	High
Pow 2018^56^	Canada	Cohort	29	General public adverts	Rheumatoid arthritis	Partner behaviours	Low
Rabbitts 2015^59^	United States	Cohort	60	Inpatient hospital	Postsurgical pain	Parent cognitions	Moderate
Rabbitts 2020^58^	United States	Cohort	119	Inpatient hospital	Postsurgical pain	Parent cognitions	Low
Rabey 2022^60^	Australia	Cohort	262	General public adverts	Chronic low back pain	Partner behaviours	Moderate
Ramchandani 2006^61^	United Kingdom	Cohort	8272	General public cohort	Abdominal pain	Parent mental health	High
Rosenbloom 2021^63^	Canada	Cohort	79	Inpatient hospital	Postsurgical pain	Parent mental health Parent cognitions	High
Rossi 2020^64^	Canada	Cohort	89	Outpatient clinics	Pelvic pain	Partner perceptions of patient self-efficacy	High
Sanders 1996^65^	Australia	RCT	44	Outpatient clinic	Abdominal pain	Parent behaviours	High
Schmaling 2020^66^	United States	Cohort	68	Outpatient clinic	Fibromyalgia	Partner behaviours	High
Siemer 2020^69^	United States	Cohort	95	Inpatient hospital	Postsurgery pain	Parent cognitions	High
Soltani 2022^72^	Canada	Cohort	156	Outpatient pain management	Variety of chronic pain conditions	Parent cognitions	High
Sorbi 2006^73^	The Netherlands	Cohort	80	General public adverts	Any chronic pain	Partner behaviours	High
Stone 2017^76^	United States	Cohort	153	Outpatient clinic	Functional abdominal pain	Parent behaviours	Low
Stone 2018^74^	United States	Cohort	160	Outpatient clinic	Functional abdominal pain	Parent behaviours	Low
Stone 2020^75^	United States	Cohort	278	Outpatient clinic	Functional abdominal pain	Parent mental health Parent cognitions Parent behaviours	High
Timko 1993^78^	United States	Cohort	204	Outpatient clinic	Arthritis and other rheumatic disease	Parent mental health	High
Welkom 2013^82^	United States	Cohort	121	Outpatient clinic	MSK pain	Parent cognitions Parent behaviours	Moderate
Wickrama 2023^83^	United States	Cohort	11,030	General public cohort	Variety of chronic pain conditions	Parent behaviours	High
Woods 2019^84^	United States	Cohort	3495	General public adverts	Any chronic pain	Partner support	High

Studies investigated 2 dyads:

(1) Parents and children: 38 studies, 27,814 dyads.
(2) Partners: 14 studies, 4904 dyads.

There were a variety of study designs across studies:

(1) Cohorts: 46 studies.
(2) Randomised controlled trials: 2 studies.
(3) Experimental: 2 studies.
(4) Ecological momentary assessment: 2 studies.

Of the included studies, the majority clearly reported controlling for potential confounding factors (41/52). The most common variables controlled for were youth age and sex. In total, 25 studies also controlled for pain-related factors, or factors theoretically linked to pain.

**Figure 2.**
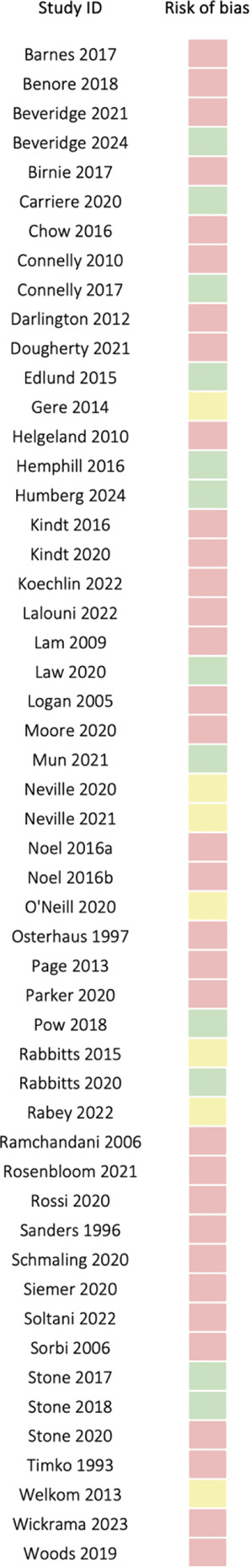
Risk-of-bias ratings. Red, high risk of bias; yellow, moderate risk of bias; green, low risk of bias.

Most studies were rated as high risk of bias (33/52). Seven were rated as moderate risk of bias and 12 as low risk of bias. A visual representation is given in Figure 2. For parent studies, 26 of 38 studies were high risk of bias (68%), 4 were moderate risk of bias (11%), and 8 were low risk of bias (21%). For partner studies, 7 were high risk of bias (50%), 3 were moderate risk of bias (21%), and 4 were low risk of bias (29%). The majority of high-risk studies were cohort studies undertaking secondary data analysis of existing databases or studies. These studies often insufficiently reported recruitment and analysis information, such as attrition (rates, reasons for withdrawal) and methods for dealing with missing data.

Studies investigated a range of interpersonal mechanisms (some studies investigated multiple mechanisms).

(1) Parents:
  Parental mental health: 17 studies.
  Parental cognitions: 17 studies.
  Parental behaviours: 21 studies.
(2) Partners:
  Partner support behaviours: 9 studies.
  Relationship satisfaction: 2 studies.
  Partner mental health: 2 studies.
  Partner confidence: 2 studies.
  Partner perceptions of patient self-efficacy: 1 study.
  Partner validation: 1 study.
  Goal conflict between couples: 1 study.
  Partner expressed emotion: 1 study.

GRADE judgments for the certainty of the evidence for all mechanisms are available in the supplemental digital content (http://links.lww.com/PAIN/C225).

### 3.3. Excluded studies

We excluded 211 studies throughout the course of this review. Most studies were excluded for not reporting the effect of a dyadic variable on a pain outcome. For example, mechanisms were individual (eg, pain self-efficacy) or societal (eg, socioeconomic status). We also excluded 54 studies that did not include outcomes related to pain or pain-related function, and 41 studies that were cross-sectional. Other reasons for exclusion are given in Figure 1.

### 3.4. Parent and child results

Three groups of predictive factors were identified across the parent and child studies: parent mental health, parent cognitions, and parent behaviours. The evidence for each of these mechanisms is summarised below. Study results are available in summary of findings tables (Tables 2–4).

**Table 2:** Parent mental health summary of findings.

	Parent mental health										
	Study ID	Study design	Dyads (n)	Child age (range and mean, SD)	% Mothers	Duration (baseline to follow-up)	Mental health subtype	Child pain outcome	Effect*	Transition	QA rating
Anxiety	Barnes 2017^1^	Cohort	81	3 y-17 y and 8 mo Mean age 10.5 (4.7)	79.0	Length of hospital stay (median 4.2 d)	General anxiety	Pain intensity	▲	Worsening	High
	Beveridge 2024^5^	Cohort	76	10-18 y	89.5	7 d	General anxiety	Pain intensity	▭	No effect	
							Anxiety variability	Pain intensity	▭	No effect	
							General anxiety	Pain interference	▲	Worsening	
							Anxiety variability	Pain interference	▭	No effect	
	Darlington 2012^14^	Cohort	2230	10-12 y	95.6	2-2.5 y	Maternal general anxiety	Presence of chronic pain	▲	Onset	High
							Maternal general anxiety	Presence of severe chronic pain	▲	Onset	
	Helgeland 2010^25^	Cohort	456	18 mo at T1 12 y at T2	100	10 y	General anxiety	Presence of chronic pain	▭	No effect	High
	Humberg 2024^28^	Cohort	689	Mean age 13.44 (1.84)	NR	1 y	General anxiety	Pain subgroups	▭	No effect	
	Page 2013^51^	Cohort	83	8-18 y Mean age 13.8 (2.4)	75.9	2 wk	Pain anxiety	Pain intensity	▲	Worsening	High
						6 mo	Pain anxiety	Pain intensity	▭	No effect	
						12 mo	Pain anxiety	Pain intensity	▭	No effect	
	Ramchandani 2006^61^	Cohort	8272	6 y 9 mo	95.6% had data from mothers 64.8% data from fathers	6 y	Maternal anxiety	Presence of chronic pain	▲	Onset	High
							Maternal anxiety dose-response	Presence of chronic pain	▲	Onset	
							Paternal anxiety	Presence of chronic pain	▲	Onset	
							Paternal anxiety dose-response	Presence of chronic pain	▲	Onset	
	Rosenbloom 2021^63^	Cohort	79	9-18 y Mean age 14.56 (2.31)	84.7	12 mo	Pain anxiety	Pain interference	▲	Worsening	High
							General anxiety	Pain interference	▲	Worsening	
							Pain anxiety	Functional disability	▲	Worsening	
							General anxiety	Functional disability	▲	Worsening	
	Stone 2020^75^	Cohort	278	11-17	92	7 d	General anxiety	Pain subgroups	▲	Worsening	High
Depression	Barnes 2017^1^	Cohort	81	3 y-17 y and 8 mo Mean age 10.5 (4.7)	79.0	Length of hospital stay (median 4.2 d)	Depression	Pain intensity	▲	Worsening	High
	Beveridge 2021^6^	Cohort	192	10-18 y Mean age 14.38 (2.2)	92.2	3 mo	Depression	Pain interference	▭	No effect	High
	Beveridge 2024^5^	Cohort	76	10-18 y	89.5	7 d	Low mood	Pain intensity	▭	No effect	
							Low mood variability	Pain intensity	▭	No effect	
							Low mood	Pain interference	▭	No effect	
							Low mood variability	Pain interference	▭	No effect	
	Darlington 2012^14^	Cohort	2230	10-12 y	95.6	2-2.5 y	Maternal depression	Presence of chronic pain	▭	No effect	High
							Maternal depression	Presence of severe chronic pain	▭	No effect	
	Helgeland 2010^25^	Cohort	456	18 mo at T1 12 y at T2	100	10 y	Maternal depression	Presence of chronic pain	▲	Onset	High
	Humberg 2024^28^	Cohort	689	Mean age 13.44 (1.84)	NR	1 y	Depression	Pain subgroups	▭	No effect	
	Moore 2020^41^	Cohort	50	6 y	94.0	5 y	Maternal depression	Chronic pain frequency	▭	No effect	High
	Mun 2021^42^	Cohort	731	2 y-2 y 11 mo	NR	12 y	Depression	Pain symptom trajectories	▭	No effect	Low
	Neville 2020^43^	Cohort	95	10-18 y Mean age 14.08	94.5	7 d	Negative mood	Pain intensity	▲	Worsening	Moderate
	Ramchandani 2006^61^	Cohort	8272	6 y 9 mo	95.6% had data from mothers 64.8% data from fathers	6 y	Maternal depression	Presence of chronic pain	▭	No effect	High
							Paternal depression	Presence of chronic pain	▭	No effect	
	Rosenbloom 2021^63^	Cohort	79	9-18 y Mean age 14.56 (2.31)	84.7	12 mo	Depression	Pain interference	▭	No effect	High
							Depression	Functional disability	▭	No effect	
	Stone 2020^75^	Cohort	278	11-17	92	7 d	Depression	Pain subgroups	▲	Worsening	High
	Timko 1993^78^	Cohort	204	Mean age 9.3 y	NR	4 y	Maternal depression	Pain intensity	▭	No effect	High
							Paternal depression	Pain intensity	▭	No effect	
							Maternal depression	Functional disability	▭	No effect	
							Paternal depression	Functional disability	▲	Worsening	
Distress	Helgeland 2010^25^	Cohort	456	18 mo at T1 12 y at T2	100	10 y	Maternal distress	Presence of chronic pain	▲	Worsening	High
	Logan 2005^39^	Cohort	78	7-17 y Mean age 12.1 (2.9)	96.2	3 wk	Maternal distress	Functional disability	▲	Worsening	High
							Paternal distress	Functional disability	▭	No effect	
Stress	Beveridge 2024^5^	Cohort	76	10-18 y	89.5	7 d	Parenting stress	Pain intensity	▭	No effect	
							Parenting stress variability	Pain intensity	▭	No effect	
							Parenting stress	Pain interference	▲	Worsening	
							Parenting stress variability	Pain interference	▭	No effect	
	Darlington 2012^14^	Cohort	2230	10-12 y	95.6	2-2.5 y	Maternal stress	Presence of chronic pain	▲	Onset	High
							Maternal parenting stress	Presence of chronic pain	▲	Onset	
							Maternal stress	Presence of severe chronic pain	▲	Onset	
							Maternal parenting stress	Presence of severe chronic pain	▲	Onset	
Personal strain	Timko 1993^78^	Cohort	204	Mean age 9.3 y	NR	4 y	Maternal personal strain	Pain intensity	▭	No effect	High
							Paternal personal strain	Pain intensity	▭	No effect	
							Maternal personal strain	Functional disability	▲	Worsening	
							Paternal personal strain	Functional disability	▭	No effect	
Hope	Moore 2020^41^	Cohort	50	6 y	94.0	5 y	Maternal hope	Chronic pain frequency	▼	Improving	High
Psychosocial	Benore 2018^2^	Cohort	670	Mean age 15.4 (2.8)	NR	6 mo	Psychosocial functioning (baseline)	Pain intensity	▭	No effect	High
							Psychosocial functioning (change)	Pain intensity	▼	Improving	
PTSD	Li 2022^37^	Cohort	1139	Mean age: 22.5 (10.28)	NR	NR	PTSD	Presence of chronic pain	▲	Onset	Moderate

* Effect symbols represent: ▲ = mechanisms is significantly positively associated with outcome; ▼ = mechanism is significantly negatively associated with outcome; ▭ = no significant effect of mechanism on outcome.

**Table 3:** Parent cognitions summary of findings.

	Parent cognitions										
	Study ID	Study design	Dyads (n)	Child age (y) (range and mean, SD)	% Mothers	Duration (baseline to follow-up)	Cognition subtype	Child pain outcome	Effect*	Transition	QA risk rating
Catastrophizing	Birnie 2017^7^	Cohort	167	10-20 M = 14.55 (1.82)	89.5	Variable—presurgery to follow-up Mean length 6.5 wks	Pain catastrophizing about child's pain (presurgery)	Pain intensity	▭	No effect	High
	Chow 2016^10^	Cohort	195	8-19 M = 13.8 (2.42)	94.4	4 mo	Pain catastrophizing about child's pain—magnification and rumination	Functional disability	▭	No effect	High
							Pain catastrophizing about child's pain—helplessness	Functional disability	▭	No effect	High
							Fear of pain	Functional disability	▭	No effect	High
	Dougherty 2021^16^	Cohort	155	8-17 M = 13.95 (2.39)	NR	6 mo and 12 mo	Pain catastrophizing about child's pain	Functional disability	▲	Worsening	High
	Lalouni 2022^34^	Cohort	90	8-12	86	10 wk	Parent catastrophizing about child's pain (mediation analysis)	Pain severity	▲	Worsening	
	Law 2020^36^	Cohort	239	11-17 M = 14.7 (1.9)	94	6 mo	Pain catastrophizing about child's pain	Headache frequency	▲	Worsening	Low
							Pain catastrophizing about child's pain	Headache related disability	▲	Worsening	Low
	Neville 2020^43^	Cohort	95	10-18 M = 14.08	94.5	7 d	Pain catastrophizing about child's pain	Pain interference	▲	Worsening	Moderate
	Neville 2021^44^	Cohort	152	10-18 M = 14.23 (2.25)	93	3 mo	Pain catastrophizing about child's pain	Pain interference	▭	No effect	Moderate
							Intolerance of uncertainty	Pain interference	▭	No effect	Moderate
	Page 2013^51^	Cohort	83	8-18 M = 13.8 (2.4)	75.9	2 wk	Pain catastrophizing (postsurgery)	Pain intensity	▭	No effect	High
						6 mo	Pain catastrophizing (postsurgery)	Pain intensity	▭	No effect	High
						12 mo	Pain catastrophizing (postsurgery)	Pain intensity	▲	Onset	High
	Parker 2020^53^	Cohort	113	8-18 M = 14.41 (2.38)	88.5	1 mo	Pain catastrophizing about child's pain	Pain interference	▼	Improving	High
	Rabbitts 2015^59^	Cohort	60	10-18 M = 14.7 (1.9)	NR	12 mo	Pain catastrophizing about child's pain (presurgery)	Pain resolution	▲	Worsening	Moderate
	Rabbitts 2020^58^	Cohort	119	10-18 M = 14.9	87.4	2 wk	Pain catastrophizing about child's pain (presurgery)	Presence of acute pain	▭	No effect	Low
						4 mo	Pain catastrophizing about child's pain (presurgery) Univariate analysis	Presence of chronic pain	▲	Onset	Low
						4 mo	Pain catastrophizing about child's pain (presurgery) Multivariate analysis + youth psychosocial factors	Presence of chronic pain	▭	No effect	Low
	Rosenbloom 2021^63^	Cohort	79	9-18 M = 14.56 (2.31)	84.7	12 mo	Pain catastrophizing about child's pain (presurgery)	Pain interference	▭	No effect	High
							Pain catastrophizing about own pain (presurgery)	Pain interference	▭	No effect	High
							Pain catastrophizing about child's pain (presurgery)	Functional disability	▭	No effect	High
							Pain catastrophizing about own pain (presurgery)	Functional disability	▭	No effect	High
	Siemer 2020^69^	Cohort	76	10-17	79	12 mo	Pain catastrophizing about child's pain Presurgery Univariate analyses	Pain interference	▲	Onset	High
							Pain catastrophizing about child's pain Presurgery Multivariate analyses + child symptom profile	Pain interference	▭	No effect	High
	Stone 2020^75^	Cohort	278	11-17	92	7 d	Pain catastrophizing about child's pain	Pain subgroups	▲	Worsening	High
Intolerance of uncertainty	Neville 2021^44^	Cohort	152	10-18 M = 14.23 (2.25)	93	3 mo	Intolerance of uncertainty	Pain interference	▭	No effect	Moderate
	Soltani 2022^72^	Cohort	156	10-18 M = 14.27	90.3	3 mo	Intolerance of uncertainty	Pain interference	▭	No effect	High
Cognitive reappraisal	Koechlin 2022^33^	Cohort	56	11-17 M = 14.5 (1.9)	92.9	2 wk	Cognitive reappraisal	Pain interference	▭	No effect	High

* Effect symbols represent: ▲ = mechanism is significantly positively associated with outcome; ▼ = mechanism is significantly negatively associated with outcome; ▭ = no significant effect of mechanism on outcome.

**Table 4:** Parent behaviours summary of findings.

	Parent behaviours										
	Study ID	Study design	Dyads (n)	Child age (y) (range and mean, SD)	% Mothers	Duration (baseline to follow-up)	Behaviour subtype	Child pain outcome	Effect*	Transition	QA rating
	Chow 2016^10^	Cohort	195	8-19 M = 13.8 (2.42)	94.4	4 mo	Protective behaviours	Functional disability	▭	No effect	High
	Connelly 2010^11^	Ecological momentary assessment	9	8-16 M = 12.3 (3.4)	88.9	2 wk	Protective behaviours	Activity limitations due to pain	▲	Worsening	High
	Connelly 2017^12^	Ecological momentary assessment	66	7-18 M = 12.8 (2.8)	86	1 mo	Protective behaviours	Pain intensity	▲	Worsening	Low
							Protective behaviours	Pain interference	▲	Worsening	
	Dougherty 2021^16^	Cohort	155	8-17 M = 13.95 (2.39)	NR	6 mo and 12 mo	Protective behaviours	Functional disability	▲	Worsening	High
	Beveridge 2024^5^	Cohort	76	10-18 y	89.5	7 d	Protective behaviours	Pain intensity	▭	No effect	
							Protective behaviours variability	Pain intensity	▭	No effect	
							Protective behaviours	Pain interference	▲	Worsening	
							Protective behaviours variability	Pain interference	▭	No effect	
	Lalouni 2022^34^	Cohort	90	8-12	86	10 wk	Protective behaviours (mediation analysis)	Pain severity	▭	No effect	
	Law 2020^36^	Cohort	239	11-17 M = 14.7 (1.9)	94	6 mo	Protective behaviours	Pain frequency	▲	Worsening	Low
							Protective behaviours	Pain-related disability	▲	Worsening	
	Neville 2020^43^	Cohort	95	10-18 M = 14.08	94.5	7 d	Protective behaviours	Pain unpleasantness	▲	Worsening	Moderate
							Protective behaviours	Pain interference	▲	Worsening	
							Protective behaviours	Pain intensity	▲	Worsening	
	Neville 2021^44^	Cohort	152	10-18 M = 14.23 (2.25)	93	3 mo	Protective behaviours	Pain intensity	▲	Worsening	Moderate
							Protective behaviours	Pain interference	▲	Worsening	
	Noel 2016^46^ [child study]	Cohort	89	7-11 M = 9.28 (1.26)	94.4	4 wk	Protective behaviours (change)	Pain intensity	▭	No effect	Low
							Protective behaviours (change)	Functional disability	▭	No effect	
	Noel 2016^46^ [adolescent study]	Cohort	241	12-17 M = 14.97 (1.34)	92.5	12 wk	Protective behaviours (change)	Pain intensity	▼	Improvement	Low
							Protective behaviours (change)	Functional disability	▭	No effect	
	Welkom 2013^82^	Cohort	121	11-18 M = 15.58 (1.53)	91.7	2 mo	Protective behaviours (change) (adolescent-reported)	Functional disability	▭	No effect	Moderate
							Protective behaviours (change) (parent-reported)	Functional disability	▭	No effect	
	Connelly 2017^12^	Ecological momentary assessment	66	7-18 M = 12.8 (2.8)	86	1 mo	Minimising responses	Pain intensity	▭	No effect	Low
							Minimising responses	Pain interference	▲	Worsening	
	Noel 2016^46^ [child study]	Cohort	89	7-11 M = 9.28 (1.26)	94.4	4 wk	Minimising responses (change)	Pain intensity	▭	No effect	Low
							Minimising responses (change)	Functional disability	▭	No effect	
	Noel 2016^46^ [adolescent study]	Cohort	241	12-17 M = 14.97 (1.34)	92.5	12 wk	Minimising responses (change)	Pain intensity	▭	No effect	Low
							Minimising responses (change)	Functional disability	▭	No effect	
	Connelly 2017^12^	Ecological momentary assessment	66	7-18 M = 12.8 (2.8)	86	1 mo	Monitoring responses	Pain intensity	▭	No effect	Low
							Monitoring responses	Pain interference	▭	No effect	
	Lalouni 2022^34^	Cohort	90	8-12	86	10 wk	Monitoring responses (mediation analysis)	Pain severity	▭	No effect	
	Noel 2016^46^ [child study]	Cohort	89	7-11 M = 9.28 (1.26)	94.4	4 wk	Monitoring responses (change)	Pain intensity	▭	No effect	Low
							Monitoring responses (change)	Functional disability	▭	No effect	
	Noel 2016^46^ [adolescent study]	Cohort	241	12-17 M = 14.97 (1.34)	92.5	12 wk	Monitoring responses (change)	Pain intensity	▭	No effect	Low
							Monitoring responses (change)	Functional disability	▭	No effect	
	Connelly 2017^12^	Ecological momentary assessment	66	7-18 M = 12.8 (2.8)	86	1 mo	Distracting responses	Pain intensity	▭	No effect	Low
							Distracting responses	Pain interference	▭	No effect	
	Noel 2016 [child study]^46^	Cohort	89	7-11 M = 9.28 (1.26)	94.4	4 wk	Distracting behaviours (change)	Pain intensity	▭	No effect	Low
							Distracting behaviours (change)	Functional disability	▭	No effect	
	Noel 2016^46^ [adolescent study]	Cohort	241	12-17 M = 14.97 (1.34)	92.5	12 wk	Distracting behaviours (change)	Pain intensity	▭	No effect	Low
							Distracting behaviours (change)	Functional disability	▭	No effect	
	Noel 2016^46^ [child study]	Cohort	89	7-11 M = 9.28 (1.26)	94.4	4 wk	Solicitous behaviours (change)	Pain intensity	▭	No effect	Low
							Solicitous behaviours (change)	Functional disability	▭	No effect	
	Noel 2016^46^ [adolescent study]	Cohort	241	12-17 M = 14.97 (1.34)	92.5	12 wk	Solicitous behaviours (change)	Pain intensity	▼	Improvement	Low
							Solicitous behaviours (change)	Functional disability	▭	No effect	
	Stone 2020^75^	Cohort	278	11-17	92	7 d	Protective and solicitous behaviours	Pain symptom trajectories	▲	Worsening	High
	Connelly 2010^11^	Ecological momentary assessment	9	8-16 M = 12.3 (3.4)	88.9	2 wk	Distracting/minimising responses	Activity limitations because of pain	▭	No effect	High
	Mun 2021^42^	Cohort	731	2 at baseline 14 at follow-up	NR	12 y	Harsh parenting styles	Pain symptom trajectories	▭	No effect	Low
	Sanders 1996^65^	Cohort	43	7-14 M = 9.42 (2.04)	100	6 mo	Maternal adaptive caregiving	Pain intensity (change)	▲	Improving	High
							Maternal maladaptive caregiving	Pain intensity (change)	▭	No effect	
							Maternal adaptive caregiving	Pain interference	▭	No effect	
							Maternal maladaptive caregiving	Pain interference	▭	No effect	
	Osterhaus 1997^49^	Cohort	39	12-22	NR	4 wk	Maternal encouragement of illness behaviour (mother report)	Reduction in pain symptoms	▭	No effect	High
						4 wk	Maternal encouragement of illness behaviour (child report)	Reduction in pain symptoms	▼	Worsening	
						4 wk	Paternal encouragement of illness behaviours (father report)	Reduction in pain symptoms	▭	No effect	
						4 wk	Paternal encouragement of illness behaviours (child report)	Reduction in pain symptoms	▭	No effect	
						1 y	Maternal encouragement of illness behaviour (mother report)	Reduction in pain symptoms	▭	No effect	
						1 y	Maternal encouragement of illness behaviour (child report)	Reduction in pain symptoms	▼	Worsening	
						1 y	Paternal encouragement of illness behaviours (father report)	Reduction in pain symptoms	▭	No effect	
						1 y	Paternal encouragement of illness behaviours (child report)	Reduction in pain symptoms	▭	No effect	
	Stone 2017^76^	Cohort	138	11-17 M = 14.2 (1.82)	92	7 d	Parent pain behaviours (adolescent-reported)	Pain intensity	▲	Worsening	Low
							Parent pain behaviours (adolescent-reported)	Pain bothersomeness	▲	Worsening	
							Parent pain behaviours (adolescent-reported)	Number of pain locations	▲	Worsening	
	Stone 2020^75^	Cohort	278	11-17	92	7 d	Parent pain behaviours (parent-reported)	Pain symptom trajectories	▲	Worsening	High
	Chow 2016^10^	Cohort	195	8-19 M = 13.8 (2.42)	94.4	4 mo	Parental avoidance of activities	Functional disability	▭	No effect	High
	Wickrama 2023^83^	Cohort	11,030	NR	NR	13 y	Parental rejection	Presence of chronic pain	▲	Onset	
	Koechlin 2022^33^	Cohort	56	11-17 M = 14.5 (1.9)	92.9	2 wk	Parent emotional expression	Pain interference	▼	Worsening	High

* Effect symbols represent: ▲ = mechanism is significantly positively associated with outcome; ▼ = mechanism is significantly negatively associated with outcome; ▭ = no significant effect of mechanism on outcome.

#### 3.4.1. Parent mental health

Seventeen studies (14,230 dyads) investigated parent mental health mechanisms on child pain. In total, 7 mental health mechanisms were investigated: anxiety, depression, distress, stress, personal strain, hope, and psychosocial functioning. Only anxiety, depression, distress, and stress had evidence from 2 or more studies and are therefore summarised narratively below. All studies were cohort studies, and for most studies, the majority of responses were from mothers. A summary of all study results for all mental health mechanisms is given in Table 2.

##### 3.4.1.1. Parent anxiety

Nine studies (12,244 dyads) investigated parental anxiety.^1,5,14,25,28,51,61,63,75^ Parent anxiety was measured using a range of scales (both state and trait anxiety); no analyses used clinical anxiety diagnoses.

Eight studies investigated general anxiety,^1,5,14,25,28,61,63,75^ with 6 of 8 studies finding that higher levels of parental anxiety at baseline predicted worse outcomes in child pain at follow-up.^1,5,14,61,63,75^ Two studies found no significant effect of parental anxiety on the child pain outcomes at follow-up.^25,28^

Two studies investigated the effect of parent pain anxiety on child pain outcomes using the Pain Anxiety Symptoms Scale (PASS-20^50,63^; Page (2013^51^) found an effect of pain anxiety on child pain severity, but only at 2 weeks follow-up; there was no significant associations at 6- or 12-month follow-up. In comparison, Rosenbloom (2021^63^) found a significant effect of pain anxiety on child pain interference at 12 months.

The following evidence for the effect of parental anxiety on pain transitions has been identified:

(1) Onset of pain: 2 studies (10,502 dyads) found that higher levels of parental anxiety were associated with the onset of pain.^14,61^
(2) Worsening of pain: 3 studies (243 dyads) found that higher levels of parental anxiety were associated with the worsening of pain (pain intensity and pain interference).^1,51,63^
(3) Two studies (1145 dyads) found no effect of parental pain anxiety on child pain outcomes.^25,28^

This evidence for parent anxiety is of moderate certainty.

##### 3.4.1.2. Parent depression

Thirteen studies (13,933 dyads) investigated parental depression.^1,5,6,14,25,28,41–43,61,63,75,78^ Twelve studies investigated depression,^1,5,6,14,25,28,41,42,61,63,75,78^ and one study investigated “negative mood.”^43^ Nine different measures were used across the 10 studies; no analyses used clinical depression diagnoses.

Five studies reported a significant effect of higher levels of depression on worse child pain outcomes^1,25,43,75,78^; however, in the study by Timko (1993), only one analysis out of 4 was significant. The remaining 8 studies reported no significant effect of parental depression on child pain outcomes.^5,6,14,28,41,42,61,63^

The following evidence for the effect of parental depression on pain transitions has been identified:

(1) Onset of pain: one study (456 dyads) found that higher levels of parental depression were associated with the onset of pain.^25^
(2) Worsening of pain: 5 studies (1114 dyads) found that higher levels of parental depression were associated with worse child pain outcomes at follow-up (pain intensity and functional disability).^1,25,43,75,78^
(3) Eight studies (12,321 dyads) found no significant effect of parental depression on child pain outcomes.^5,6,14,28,41,42,61,63^

This evidence for parent depression is of low certainty.

##### 3.4.1.3. Parent distress

Two studies (534 dyads) investigated parental distress, defined as combined symptoms of anxiety and depression.^25,39^ Both studies found evidence of a significant effect of maternal distress on worse child pain outcomes (presence of chronic pain and functional disability), indicating a worsening pain transition; however, there was no evidence of a significant effect for paternal distress. This evidence for parent distress is of very low certainty.

##### 3.4.1.4. Parenting stress

Two studies (2308 dyads) investigated parenting stress.^5,14^ Darlington (2012^14^) found significant effects of maternal and paternal parenting stress on the onset of child pain; Beveridge (2024^5^) found a significant effect of parenting stress on child pain interference (worsening pain transition) but not child pain intensity. This evidence is of very low certainty.

#### 3.4.2. Parent cognitions

Seventeen studies (2135 dyads) investigated parent cognitions on child pain. Four cognitive mechanisms were investigated: pain catastrophizing, intolerance of uncertainty, fear of pain, and cognitive reappraisal. Only pain catastrophizing and intolerance of uncertainty had evidence from 2 or more studies and are therefore reported narratively below. A summary of all study results for all cognitive mechanisms is given in Table 3.

##### 3.4.2.1. Parent catastrophizing

Fourteen studies (1654 dyads) investigated the effect of parent pain catastrophizing on child pain outcomes.^7,10,16,34,36,43,44,51,53,58,59,63,69,75^ All studies were cohort studies and used the Pain Catastrophizing Scale as the measure, either the original version or the parent version.

Overall, results were mixed. All studies reported multiple analyses to investigate the effect of catastrophizing on various pain outcomes. Ten studies reported an analysis in which greater parental pain catastrophizing was associated with the onset of pain or worse child pain outcomes.^16,34,36,43,51,58,59,69,75^ However, 7 studies reported an analysis in which there was no statistically significant effect of parental pain catastrophizing on child pain outcomes.^7,10,44,51,58,63,69^ One study reported that change in parent catastrophizing from baseline to follow-up (less catastrophizing) was associated with an improvement in child pain interference.^53^ As is evident, 3 of these studies reported mixed results, with significant effects for some pain-related outcomes and no effects for others.^51,58,69^

Six studies (584 dyads) investigated the effect of parental pain catastrophizing on postsurgical pain.^7,51,58,59,63,69^ Five studies examined parent catastrophizing before surgery.^7,58,59,63,69^ Analyses from 3 of these studies found no effect of catastrophizing on pain^7,58,63^ at 6.5-week, 2-week, and 12-month follow-ups, respectively. By contrast, 3 analyses found a statistically significant effect of catastrophizing on the onset of postsurgical pain^58,59,69^ at 4-month and 12-month follow-ups. However, for 2 of these significant onset analyses,^58,69^ when youth psychosocial factors and child symptoms profiles were included in the multivariate analyses, the effect of parent catastrophizing became nonsignificant. Only one study investigated the effect of parental catastrophizing after child surgery on postsurgical pain,^51^ finding no effect at 2-week or 6-month follow-up, but a significant effect for worse pain intensity at 12 months.

Three studies explored whether there were interactions between parent and child catastrophizing.^7,16,53^ Although Dougherty (2021^16^) found a significant association, such that higher levels of parent catastrophizing led to higher levels of child catastrophizing, no significant associations were found by Birnie (2017^7^) or Parker (2020^53^). However, Birnie (2017^7^) did not find any evidence of partner effects (child on parent and vice versa), but there were significant actor effects such that baseline child and parent catastrophizing significantly predicted follow-up child and parent catastrophizing, respectively.

The following evidence for the effect of parental pain catastrophizing on pain transitions has been identified:

(1) Onset of pain: 3 studies (281 dyads) found that parent pain catastrophizing was significantly associated with the onset of child pain for postsurgical pain, particularly at longer follow-ups.^50,58,69^
(2) Worsening of pain: 5 studies (857 dyads) found that higher levels of parental catastrophizing were associated with worse child pain outcomes (functional disability, pain frequency, pain interference, pain symptoms) at follow-up.^16,34,36,43,75^
(3) Four studies (593 dyads) found no significant effect of parental catastrophizing on any child pain outcomes.^7,10,44,63^

The evidence for parent catastrophizing is of very low certainty.

##### 3.4.2.2. Parent intolerance of uncertainty

Two studies (308 dyads) investigated the effect of parental intolerance of uncertainty (individual reactions to uncertain and ambiguous situations and the future) on child pain outcomes.^44,72^ Neither study found an effect for intolerance of uncertainty on child pain.

Neville (2021^44^) undertook further analyses using structural equation modelling to examine interactions between parent and child factors in the prediction of child pain interference at 3 months. In this model, they found a significant effect of parent intolerance of uncertainty, such that greater parent intolerance of uncertainty (baseline) predicted greater pain catastrophizing (baseline), which in turn predicted greater parent protectiveness (at baseline) and subsequently greater youth fear of pain. Youth fear of pain then predicted increases over time in youth pain interference, over and above baseline pain interference.

The evidence for parent intolerance of uncertainty is of very low certainty.

#### 3.4.3. Parental behaviours

Twenty-one studies (2141 dyads) investigated parent behaviour mechanisms on child pain. Parent behaviours included both parent behaviours directed towards the child, and parent behaviours in relation to themselves. Eleven behaviour mechanisms were investigated: protective behaviours, minimising responses, monitoring responses, distracting responses, solicitous behaviours, harsh parenting styles, adaptive and maladaptive caregiving, encouragement of illness behaviours, parent pain behaviours, parent avoidance of activities, and parent emotional expression. Seventeen studies were cohort studies (5 were data from randomised controlled trials); and 2 studies were ecological momentary assessments. An overview of all studies and results is given in Table 4.

##### 3.4.3.1. Parent behaviours towards children

###### 3.4.3.1.1. Parent protective behaviours

Twelve studies (1528 dyads) investigated parent protective behaviours.^5,11,12,13,17,35,37,43,44,46,82^ All used the Adult Responses to Child Symptoms questionnaire protect subscale, which defines protective behaviours as the “provision of special attention or privileges, or assisted reduction of normal responsibilities in response to the expression of pain or potential for pain.”^47^ All studies used parent self-reported behaviours, apart from one study that also included adolescent reports of parental protective behaviours.^82^

Evidence was mixed; although 6 studies found that parent protective behaviours were significantly associated with worse child pain outcomes at follow-up,^11,12,16,36,43,44^ 4 studies found no statistically significant effects.^10,34,46,82^ One study with 241 dyads examined change in parent protective behaviours over the 12-week course of study, findings that a reduction in protective behaviours was associated with an improvement in pain intensity, but not functional disability.^46^ One study with 76 dyads found evidence for protective behaviours on child pain interference, but not child pain intensity.^5^ The study that investigated both parent-reported and adolescent-reported parental protective behaviours found no significant effect for either on pain-related disability.^82^

The following evidence for the effect of parental pain protective behaviours on pain transitions has been identified.

(1) Worsening of pain: Six studies (716 dyads) found that greater parent protective behaviours were significantly associated with worse child pain outcomes (activity limitations, pain intensity, pain interference, pain-related disability, pain unpleasantness) at follow-up.^11,12,16,36,43,44^
(2) Four studies (495 dyads) found no effect for parent protective behaviours on child pain outcomes (functional disability, pain severity, pain intensity).^11,35,46,82^

Overall, the evidence for parent protective behaviours is of low certainty.

###### 3.4.3.1.2. Parent minimising responses

Three studies (396 dyads) examined parent minimising responses to child pain.^13,46^ All studies used the Adult Responses to Child Symptoms questionnaire minimise subscale, which defines minimising responses as “verbal discounting of the significance of pain or criticising the expression of pain as excessive.”^47^ Only one study found evidence for a pain transition: Connelly (2017; [66 dyads]) found that greater parent minimising responses were significantly associated with worse child pain interference, but not pain intensity. Noel (2016) found no effect of minimising responses on child pain intensity or functional disability in a total of 330 dyads. The evidence for parent minimising behaviours is of low certainty.

###### 3.4.3.1.3. Parent monitoring behaviours

Four studies (486 dyads) examined parent minimising responses to child pain.^13,35,46^ All studies used parent self-report data through the Adult Responses to Child Symptoms questionnaire monitor subscale, which defines monitoring responses as “use of inquiry and extra vigilance to check on the child's pain status.”^47^ No significant effect of parent monitoring behaviours on any child pain outcomes (pain intensity, pain interference, pain severity, or functional disability) was found in any study. Therefore, no evidence for an effect of parent monitoring behaviours on any pain transitions was identified. The evidence for parent minimising behaviours is of low certainty.

###### 3.4.3.1.4. Parent distracting responses

Three studies (396 dyads) examined parent distracting responses to child pain.^13,46^ All studies used parent self-report data through the Adult Responses to Child Symptoms questionnaire distract subscale, which defines distracting responses as “efforts to engage the child's attention in other activities or sensory stimuli besides pain.”^47^ No significant effect of parent distracting behaviours on child pain outcomes (pain intensity, pain interference, or functional disability) was found in any study. Therefore, no evidence for an effect of parent monitoring behaviours on any pain transitions was identified. The evidence for parent distracting behaviours is of very low certainty.

##### 3.4.3.2. Parent self-directed behaviours

Four studies examined parent behaviours related to themselves, rather than towards the child.^10,33,75,76^ Only parent pain behaviours had more than 2 studies, reported below.

###### 3.4.3.2.1. Parent pain behaviours

Two studies (416 dyads) examined parents' own pain behaviours on child pain outcomes.^75,76^ Parent pain behaviours were defined as observable behaviours displayed by the parent that presumptively indicate that they are in pain (eg, moving slowly). Stone 2017 examined adolescents' reports of parents' behaviours, while Stone 2020 examined parents' self-reported behaviours. Both studies found significant effects, such that more frequent parent self-directed pain behaviours are associated with worse child pain intensity, pain bothersomeness, number of pain locations, and pain trajectories. This indicates that parent self-directed pain behaviours are associated with worsening child pain transitions.

Stone 2018 undertook further analyses of path models to test a model of social learning theory pathways on the relationship between parental chronic pain and adolescent chronic pain severity and functional impairment. Their model found that parent chronic pain status (baseline) was significantly associated with more frequent adolescent-reported and parent-reported parent pain behaviours (baseline). More frequent parent pain behaviours were then significantly associated with more severe adolescent pain threat appraisals (baseline). More severe adolescent pain threat appraisals (baseline) then significantly predicted adolescent pain intensity over the 7-day period.

This evidence is of very low certainty.

### 3.5. Partner results

A number of mechanisms were identified relating to the interpersonal interactions between people with pain and their partners. An overview of all mechanisms is given in the Table 5. Each mechanism will be discussed below.

**Table 5:** Partner mechanism summary of findings.

	Partner mechanisms								
	Study ID	Study design	Dyads (n)	Duration (baseline to follow-up)	Mechanism	Pain outcome	Effect*	Transition	QA rating
Behaviours	Hemphill 2016^26^	Cohort	152	12 mo	Solicitous behaviours (mediation analysis)	Physical function	▭	No effect	
	Pow 2018^56^	Cohort	29	7 d	Solicitous behaviours (patient-reported)	Pain intensity	▭	No effect	Low
					Solicitous behaviours (partner-reported)	Pain intensity	▼	Improving	
	Rabey 2022^60^	Cohort	262	1 y	Solicitous behaviours (cluster analysis)	Pain intensity	▭	No effect	Moderate
					Solicitous behaviours (cluster analysis)	Disability	▭	No effect	
					Solicitous behaviours (cluster analysis)	Pain bothersomeness	▭	No effect	
	Schmaling 2020^66^	Cohort	68	18 mo	Solicitous behaviours	Tender points	▲	Worsening	High
					Solicitous behaviours	Bodily pain	▭	No effect	
					Solicitous behaviours	Physical function	▭	No effect	
	Hemphill 2016^26^	Cohort	152	12 mo	Punishing behaviours (mediation analysis)	Physical function	▭	No effect	
	Rabey 2022^60^	Cohort	262	1 y	Punishing behaviours (cluster analysis)	Pain intensity	▭	No effect	Moderate
					Punishing behaviours (cluster analysis)	Disability	▭	No effect	
					Punishing behaviours (cluster analysis)	Pain bothersomeness	▭	No effect	
	Schmaling 2020^66^	Cohort	68	18 mo	Punishing behaviours	Tender points	▭	No effect	High
					Punishing behaviours	Bodily pain	▲	Worsening	
					Punishing behaviours	Physical function	▲	Worsening	
	Sorbi 2006^73^	EMA	80	4 wk	Punishment of pain behaviours	Pain intensity	▭	No effect	High
					Punishment of well behaviours	Pain intensity	▲	Worsening	
	Rabey 2022^60^	Cohort	262	1 y	Distracting behaviours (cluster analysis)	Pain intensity	▭	No effect	Moderate
					Distracting behaviours (cluster analysis)	Disability	▭	No effect	
					Distracting behaviours (cluster analysis)	Pain bothersomeness	▭	No effect	
	Schmaling 2020^66^	Cohort	68	18 mo	Distracting behaviours	Tender points	▭	No effect	High
					Distracting behaviours	Bodily pain	▭	No effect	
					Distracting behaviours	Physical function	▭	No effect	
	Hemphill 2016^26^	Cohort	152	12 mo	Empathic behaviours (mediation analysis)	Physical function	▭	No effect	
	Pow 2018^56^	Cohort	29	7 d	Emotional/esteem behaviours (patient-reported)	Pain intensity	▭	No effect	Low
					Emotional/esteem behaviours (partner-reported)	Pain intensity	▼	Improving	
	Sorbi 2006^73^	EMA	80	4 wk	Reinforcement of pain behaviours	Pain intensity	▲	Worsening	High
					Reinforcement of well behaviours	Pain intensity	▲	Improving	
Support	Carriere 2020^9^	Daily diary	124	7 d	Partner supportiveness	Pain intensity	▭	No effect	
	O'Neill 2020^48^	Daily diary	162	32 d	Perceived partner responsiveness	Pain intensity	▼	Improving	Moderate
	Woods 2019^84^	Cohort	3495	10 y	Partner support	Onset of chronic pain	▭	No effect	High
					Partner support	Transition from acute to chronic pain	▭	No effect	
					Partner support	Persistence of chronic pain	▭	No effect	
					Partner strain	Onset of chronic pain	▲	Onset	
					Partner strain	Transition from acute to chronic pain	▭	No effect	
					Partner strain	Persistence of chronic pain	▭	No effect	
	Gere 2014^21^	Cohort	152	6 mo	Partner perceptions of patient self-efficacy	Arthritis severity	▭	No effect	
	Hemphill 2016^26^	Cohort	152	12 mo	Partner perceptions of patient self-efficacy (mediation analysis)	Physical function	▭	No effect	
	Rossi 2020^64^	Cohort	89	3 mo	Partner perceptions of patient self-efficacy	Pain intensity	▼	Worsening	
	Kindt 2016^32^	Daily diary	70	14 d	Partner motivations to help	Pain-related disability	▭	No effect	High
	Edlund 2015^19^	Experimental	20	Pre/post experiment	Partner validation	Pain intensity	▭	No effect	Low
	Kindt 2020^31^	Experimental	68	Pre/post experiment	Goal conflict	Pain intensity (self-reported)	▲	Worsening	
					Goal conflict	Pain behaviours (observed)	▲	Worsening	
	Lam 2009^35^	Cohort	133	1 y	Partner depression	Disease severity	▲	Worsening	High
					Partner depression	Physical function	▲	Worsening	

* Effect symbols represent: ▲ = mechanism is significantly positively associated with outcome; ▼ = mechanism is significantly negatively associated with outcome; ▭ = no significant effect of mechanism on outcome.

#### 3.5.1. Partner behaviours

Five studies (591 dyads) investigated partner behaviour mechanisms on pain.^26,56,60,66,73^ Five behaviour mechanisms were explored: solicitous behaviours, punishing behaviours, distracting behaviours, emotional/empathic behaviours, and reinforcing behaviours. Only solicitous and punishing behaviours were investigated by more than 2 studies; these are summarised below.

##### 3.5.1.1. Solicitous behaviours

Partner solicitous behaviours were explored in 4 cohort studies (511 dyads). Three studies were direct analyses,^56,60,66^ and one was a mediation analysis.^26^ All studies used data provided from the person with pain, and only one study also included partner-reported data.^56^ Two scales were used: the West Haven-Yale Multidimensional Pain Inventory (WHYMPI)^26,60,66^ and the Berlin Social Support Scale.^56^ The majority of analyses found no significant effect. Only 2 analyses were significant; solicitous behaviours were significant positively associated with tender points,^66^ and partner (but not patient)-reported solicitous behaviours were significantly positively associated with a reduction in pain intensity.^56^

The following evidence for the effect of partner solicitous behaviours on pain transitions has been identified:

(1) Worsening of pain: one study (68 dyads) found an effect of partner solicitous behaviours on the number of tender pain points.^66^
(2) Improvement of pain: one study (29 dyads) found an effect for the reduction of partner solicitous behaviours resulting in lower pain intensity.^56^
(3) Four studies (511 dyads; including Refs. 56 and 66) found no statistically significant effects of partner solicitous behaviours on pain outcomes (pain intensity, pain bothersomeness, disability, or physical function), and therefore no evidence of an effect on pain transitions.^26,56,60,66^

This evidence is of very low certainty.

##### 3.5.1.2. Punishing behaviours

Punishing behaviours were examined in 4 studies (562 dyads); 3 studies were cohort studies^26,60,66^ and one was an ecological momentary assessment.^73^ All studies used data provided by the person with pain through the WHYMPI. Three studies directly investigated partner punishment of pain behaviours; 2 studies found no effect on pain outcomes,^60,73^ while one found significant effects for worsening of bodily pain and physical function, but no effect on tender points.^66^ Hemphill (2016^26^) undertook mediation analyses but found no significant effects of partner punishing responses mediating the relationship between spouse confidence in patient pain management and change in patient functional limitations at 6 or 12 months.

The following evidence for the effect of partner punishing behaviours on pain transitions has been identified.

(1) Worsening of pain: 2 studies (148 dyads) found significant effects of partner punishing behaviours on pain intensity, bodily pain, and physical function,^66,73^ indicating worsening pain transitions.
(2) Four studies (562 dyads; including Refs. 66 and 73) found no effects of partner punishing behaviours on pain intensity, pain bothersomeness, number of tender points, disability, or physical function, indicating no evidence of an effect on pain transitions^26,60,66,73^).

This evidence is of very low certainty.

#### 3.5.2. Partner support

Three studies (3771 dyads) investigated partner support mechanisms on pain. These mechanisms include partner support and partner strain (ie, lack of support). Two studies were daily diary studies, and one was a cohort study. Study durations ranged from 7 days to 10 years. Results were mixed; one study found no effect,^9^ while one found a significant negative association between support and pain intensity.^48^ The third study found no effect of partner support or partner strain on the transition from acute to chronic pain, or the persistence of chronic pain.^84^ However, there was a significant effect of partner strain on the onset of chronic pain over 10 years.

The following evidence for the effect of partner support on pain transitions has been identified.

(1) Improvement of pain: one study (162 dyads) found a significant effect of partner support on the reduction of pain intensity.^48^
(2) Two studies (3619 dyads) found no effect of partner support on the onset of chronic pain, transition of acute to chronic pain, or persistence of pain.^9,84^

This evidence is of very low certainty.

#### 3.5.3. Partner perceptions of patient self-efficacy

Three cohort studies (393 dyads) investigated partner perceptions of patient self-efficacy in managing their pain condition. Two were studies of osteoarthritis, using the Arthritis Self-Efficacy Scale,^21,26^ and one was a study of pain experienced during intercourse, which used an adapted version of the ASES—the Painful Intercourse Self-Efficacy Scale (self-efficacy for controlling pain during intercourse subscale).^64^ One study found no significant effect of partner perceptions of patient self-efficacy on disease severity over 6 months,^21^ and while one found that when couples agreed that the patient had lower levels of self-efficacy, the patient reported higher levels of pain intensity.^64^ The third study explored mediation analyses of partner perceptions of patient self-efficacy on physical function through partner empathic, solicitous, or punishing behaviours over 6 or 12 months.^26^ At 6 months, only the empathic behaviour model was significant, and at 12 months, only the solicitous behaviour model was significant. This evidence is of very low certainty.

## 4. Discussion

### 4.1. Overview of results

This review synthesized the longitudinal evidence of interpersonal psychosocial mechanisms on pain transitions in 2 dyads: parents/caregivers and children, and adults with partners. The level of certainty in the findings for most factors is low or very low certainty, with the only exception being parental anxiety, which is moderate certainty. Most studies were rated high risk of bias. Nevertheless, there are some emerging patterns of evidence that can inform current models. Of note, and in relation to all studies, the evidence relies on scales captured through self-report, usually at a single follow-up time point, and there are questions around interpretability.

#### 4.1.1. Parents and children

There is evidence that parental self-reported anxiety, especially generalised anxiety in mothers, is associated over time with the onset of chronic pain in their children, and with higher pain interference and disability. This might suggest that repeated exposure to mothers' cognitions and related behaviours that the world is a threatening and frightening place leads to alterations in the processing of pain in children, possibly through alterations in hypervigilance, avoidance, and interpretation of ambiguous physical signals as threatening and painful.^22^ However, it is also possible that mothers who live with anxiety are more likely to notice, respond to, and seek professional care for their children's symptoms. However, the absence of an association in fathers does not mean that does not exist, as this may simply reflect a sampling bias, although no specific biases were identified in this review. It is also unclear to what extent the effect of parent anxiety is direct, or indirect through family functioning. This is particularly important to explore further, as a review of cross-sectional studies of family and parent influences on paediatric chronic pain concluded that families of children with chronic pain have poorer family functioning (eg, more conflict, less cohesion) than families of healthy children.^52^ The causal and potentially reciprocal nature of the relationship between parental distress and family functioning has yet to be determined. We note the relative absence of evidence on paternal anxiety; that children's ages and follow-up times varied; and that predictors were assessed through self-report of variable reliability. In addition, although anxiety and depression overlap symptomatically, the evidence for parental depression as a unique predictor of pain transitions was contradictory.^4^ Most studies that examined parental mental health accounted for this symptomatic overlap, either by using a measure of combined distress or undertaking separate analyses. When studies examined both parental anxiety and depression in the same model, this was undertaken in multivariate models to account for interactions between the variables.

Overall, these findings generally support models of dynamic bidirectional association between parental and child responses to pain. For example, within the interpersonal fear avoidance model,^22^ there is acknowledgement that cognitive-behavioural responses in parents can affect both pain development and maintenance in a child with pain. It also highlights both direct (eg, parental overprotectiveness) and indirect (eg, social modelling) pathways, and how caring for a child with chronic pain can affect parents.

By contrast, the evidence on parental pain catastrophizing is mixed. There are several possible explanations for the contradictory evidence on catastrophizing. The concept of catastrophizing implied augmented and exaggerated worry about the consequences of the child's pain.^77^ The concept can be viewed as somewhat pejorative, as there is no “right” amount of pain for any injury or pathology; the judgement of excessive worry is unreliable; and several items on the widely used PCS^77^ are normal responses in certain contexts. Children's own expressed pain and suffering is not consistently assessed alongside parental catastrophizing but is potentially important. Where studies analysed parental catastrophizing in relation to child pain, parental catastrophizing became nonsignificant: the child's expressed pain and suffering (verbal and nonverbal) may drive both parental catastrophizing and pain outcomes.^69^

In studies that examined pain catastrophizing in parents before and after surgery, only postsurgical scores predicted worsening outcomes, suggesting that the child's suffering might be a key to the effect, rather than the anticipation of such suffering.^7^ A considerable drawback in interpreting the evidence is that catastrophizing was measured only by self-report, with no indication of behavioural expression, verbal and nonverbal, that was evident to the child.

Two studies examined general intolerance of uncertainty in parents and children; although no significant effects were found for parental intolerance of uncertainty, further analyses reported an effect of child intolerance of uncertainty on child ratings of pain interference.^72^ It is reasonable to hypothesize that intolerance of uncertainly enhances both anxiety and catastrophizing, but in the absence of quantified adequacy of explanations of the clinician's diagnosis and prognosis, and acceptance by parent or child of these explanations, the source of this uncertainty remains obscure.^55,68^ In adults, diagnostic uncertainty, defined as uncertainty in the diagnosis or aetiology of a health condition, is a predictor of patients' distress and guilt^68^, and even of cognitive processes such as recall bias.^67^ However, the studies in this review did not investigate diagnostic uncertainty, rather general intolerance of uncertainty, therefore the effect of diagnostic uncertainty is currently unknown.

Parent behaviours towards children were categorised according to the behaviours defined by the Adult Responses to Child Symptoms (ARCS) questionnaire^47,80^: protective, minimising, distracting, monitoring, and solicitous behaviours. These labels may indicate intention, but we cannot be certain that they have the effects identified, since they are defined without context. Evidence for influence of these behaviours was either of low or very low certainty. The majority of studies investigated protective behaviours, and although populations and timelines were similar, there was no consistent effect. There is also a lack of conceptual clarity in relation to protective and solicitous parental behaviours. Noel 2016b used a version of the ARCS questionnaire with a separate solicitousness subscale, although this was later found to have low reliability. Understanding whether a delineation between protective and solicitous behaviours is appropriate, or whether they are representative of the same concept is imperative to ensuring the validity and reliability of this evidence. There were few studies of other behaviours and analyses yielded no clear effects. Three studies investigated pain behaviours reported by parents who themselves had chronic pain.^74–76^ Although evidence was of very low certainty, there was a consistent effect on the worsening of child pain, but only over 7 days. Self-report of these behaviours is a weak methodology; without observational evidence that includes context, no confidence can be attached to findings. In addition, the apparent causal path between protective behaviours and poorer outcomes is susceptible to reverse causality, in which the child's expressions of suffering and reduced function lead to changes in parents' behaviours.^54^ The interaction between parents and children over time within the context of their pain, motivations, and social demands needs to be disentangled by repeated independent measures over time. We note here that the labelling of certain behaviours as “maladaptive” is simplistic and possibly judgemental, as the same behaviour from parents can be either helpful or unhelpful depending on the situation and context in which it occurs. Thus, ignoring children's pain complaints would be considered adaptive within a behavioural framework that considers attention to be a reinforcement of such behaviours. However, there is growing evidence that validating children's suffering might increase their emotional regulation, increase their ability to problem solve, reduce their anxiety, and allow them to feel more confident in re-engaging with valued but abandoned activities.

The primary model relating to the evidence base identified in this review is the Interpersonal Fear Avoidance Model of Pain (IFAM^22,70^). The IFAM posits that parent interpretations of child chronic pain (catastrophizing and pain-related fear) lead to parent engagement in maladaptive behaviours (particularly protective or minimising behaviours). These behaviours then result in the child withdrawing and avoiding activities, culminating in greater child disability. Our review identified some mechanisms within the IFAM, primarily parent pain catastrophizing, parent protective behaviours, and parent depression, although the evidence base for these is of very low certainty.

Of note, our review included parent mental health problems (depression, anxiety, distress) as predictors of child pain outcomes, rather than as consequences of children's pain, potentially consistent with the concept of “parent interpretations” in the IFAM. We propose that the model could be improved by future research capturing behaviours other than by self-report (such as expressions of pain from children, protective behaviours), and child perceptions of parents' behaviours and cognitions (such as child's perception of parental worry, rather than parent self-report of worry/catastrophizing).

#### 4.1.2. People with pain and their partners

The evidence for partner interpersonal factors on pain transitions is extremely limited. The majority of studies examining this mechanism that were identified in the screening process were either cross-sectional or did not have a pain-related outcome. We conclude that longitudinal exploration of the effects of partner support on pain has not been a priority in the quantitative research area. This is compounded by a lack of clear definitions across the entire partner chronic pain literature. Support is a vague concept, without a definitive consensus. We found that the terms “support”, “responses”, “behaviors”, “helping”, and “interactions” were all used interchangeably throughout the literature. This is a priority area for improving clarity, to ensure high-quality research is undertaken in this area.

The substantial heterogeneity in definitions of partner behaviours and support precluded any synthesis or conclusions in this review. Only 3 factors were examined by more than one study: partner behaviours, partner perceptions of patient self-efficacy, and partner support. Evidence for these factors was mixed, restricted to a small number of studies, and of very low certainty.

Partner behaviours are grouped into similar domains as in the parent and child literature: solicitous, punishing, and distracting, assessed by the self-report West Haven-Yale Multidimensional Pain Inventory (WHYMPI/MPI^30^). In a topical review of the evidence for different models describing partner/patient mechanisms, Prenevost and Reme (2017^57^) identify limitations of the operant model dominating most of the studies we describe. The model predicts that acute pain is more likely to transition to chronic through operant learning where responses from the environment positively reinforce the patient's pain behaviour and pain impact, including distress and disability. Most of the evidence is drawn from cross-sectional data but fails to consider reverse causality, whereby partners show more care where the person in pain is more distressed and disabled.^8^ Although there is some support for solicitous responses being associated with higher pain intensity and disability, there is little support for punishing and negative behaviours from partners.^57^ This is consistent with our findings, reported here, from longitudinal studies. There is also evidence that some partner behaviours were as likely to precede as to follow patient pain-related behaviour, inconsistent with operant models, and operant learning has been shown to be neither the only or even the major predictor of patient behaviour or pain.^45^

A previous review of interactions in chronic pain couples^8^ discussed the problems of models based on operant conditioning (such as solicitous behaviours) and encouraged a move towards intimacy-based research, focusing on both negative and positive factors. Studies of couple-based interventions indicate that improving partner interactions can modify pain transitions, but it is difficult to identify by what processes this occurs.^40,71^ We underline the need for research to prioritise tests of operant models vs interactional models, rather than addressing only one model. As described above, the meaning of interactions within their temporal contexts should be a clearer focus for research, and such contexts will differ between and within couples. Our Workpackage Development Group considered that validation from clinicians, demonstrating that patient suffering is believed and considered understandable, would benefit partners' understanding and flexibility in interactions. We note that, to date, there is no research linking interpersonal interactions between patients, clinicians, and partners.

### 4.2. Methodological shortcomings and future directions in interpersonal pain research

It is clear that the status quo of research into interpersonal psychosocial mechanisms for pain transitions is weak. This is despite good reasons to expect that they are important. To address this, multiple changes are required.

First, understanding the context of dyads–relationship quality, life stages, ages, sexes/genders—is critical to understanding pain. Current studies tend to amalgamate all types: for example, “parent and child” may refer to parent and baby, parent and adolescent, or even parent and adult child. We note the range of child ages in the parent and child section of this review; very few studies specified children's ages. Similarly, “partner” may refer to heterosexual or same-sex spouse or long-term significant other (which could be a parent or an adult child). Despite different expectations within such relationships, current evidence rather neglects this context, assuming the same processes, although the factors included in this review—interactions, cognitions, and behaviours, both verbal and nonverbal—are likely to differ.

Second, the dynamics of interactions have been neglected. The evidence, to date, takes self-report, usually of the frequency of behaviours, as evidence but correlation of behaviour with self-report is low,^13^ and where interaction is concerned, the observer's perception and interpretation may differ very substantially from its self-reported frequency. The dynamic nature of relationships and interactions requires multiple observers and multiple time points; we recommend more thoughtful decisions about appropriate time frames, which currently range from 7 days to 10 years.

Important gaps identified in our review include:

(1) The specific effects of parental (and partner) anxiety, distinguished from effects of family (dys)function.
(2) Possible differences between maternal and paternal anxiety and catastrophizing, in terms of expressed cognitions, perceived cognition by person with pain, changes in behaviour, and the impact on child pain transitions.
(3) Understanding the context of adaptive/maladaptive behaviours, and which situations determine whether behaviours help or not.
(4) Research on fathers; the majority of research in this review was focused on mothers.
(5) The role of diagnostic and prognostic uncertainty in the context of dyads, in respect of vulnerability to anxiety, expressions of pain, worrying and fearful cognitions about pain, and subsequent behaviours of both individuals.
(6) The role of in/validation within these interactions, both in respect of in/validation from clinicians, and from parent/partner.
(7) The effects of interpersonal dyadic mechanisms on acute pain; aside from postsurgical pain, all pain conditions in this review were chronic.
(8) Interpersonal dynamics in diverse populations including, but not limited to, those with lower socioeconomic status, non-White, non-Western, and those in developing countries.
(9) The functions and effects of protective behaviours in context, to be explored using new technologies such as sensors and electronic momentary assessment.^81^

Strengths of this review include its scope in bringing together interpersonal research across multiple dyads and the association with chronic pain. Only including longitudinal research enabled exploration of cause and effect, and allowed a more robust exploration of pain transitions that previously conducted reviews that included cross-sectional studies.^15^ However, this review was limited in its synthesis, as the substantial heterogeneity across studies precluded quantitative analysis and summary statistics. It is also limited in its exclusion of sibling and grandparent interactions, particularly important relationships in child development. The lack of independent screening and extraction was a pragmatic limitation, given the scope of the review, although rates of disagreements between reviewers was low (<5% at all stages of screening).

## 5. Conclusions

**Figure 3.**
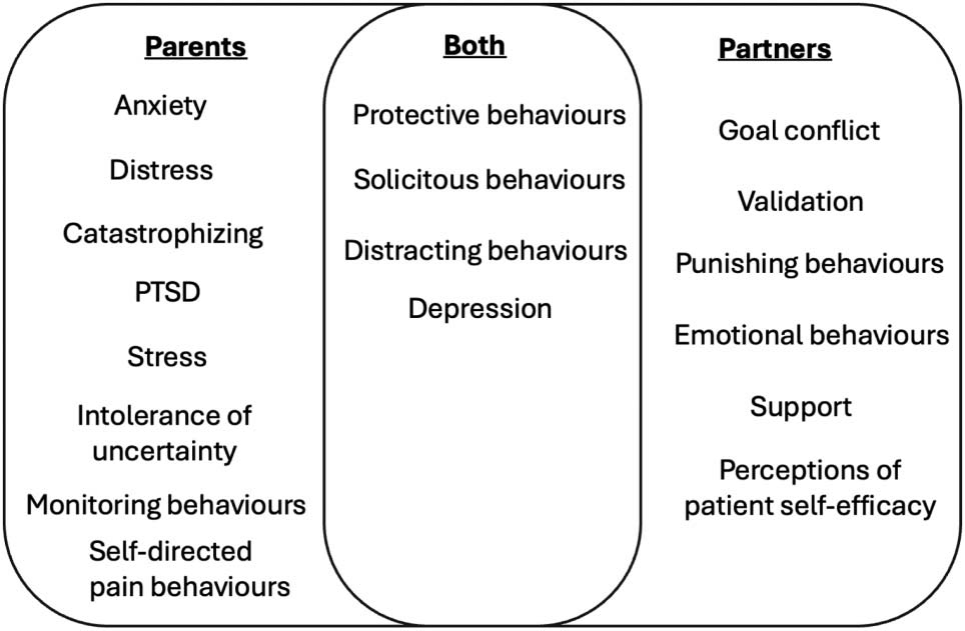
The landscape of factors studied in longitudinal interpersonal pain research.

This review has explored the potential of studying interactions between dyads of people living with pain, and how pain changes. It focused on the effect of parents and their partners, but the evidence is inconsistent and, in most studies, of low certainty because of methodological shortcomings. The strongest evidence, but only of moderate certainty, was found for parent anxiety and catastrophizing having adverse effects on pain trajectories, but even these effects were not consistent across studies. Crucial issues around context, interactions, and measurement are currently inadequately addressed. Interactions between people with pain and those they live with, or are in regular contact with, are dynamic and contextualised in the needs and lives of both people at any time point. The same cognition or behaviour in one context in one time frame could have a negative effect, while the same in a different context or time frame could have a positive effect. Therefore, context is essential in future interpersonal pain research. Currently, interpersonal research is segregated by dyads, our review highlights the lack of integration in this field, and the disparity in factors explored in parents and partners (Fig. 3). Future research would benefit from integration and exploration across dyads to establish the effects of significant others on pain transitions. Naturalistic observation and ethnographic methods have a value in extending understanding beyond operant transactions and in/validation to represent a fuller account of the life of the person in pain.

## Conflict of interest statement

E. Keogh reports unrelated consultancy services via the University of Bath to Reckitt Benckiser Health Limited. The remaining authors have no conflicts of interest to declare.

## Supplemental digital content

Supplemental digital content associated with this article can be found online at http://links.lww.com/PAIN/C225.

## Supplementary Material
