# Supplementary material for: Interpersonal dyadic influences on transitions between pain states: a narrative review and synthesis
Source: Pain. 2025 Feb 21;166(9):1974–95. doi: 10.1097/j.pain.0000000000003544 (PMC12363491; doi:10.1097/j.pain.0000000000003544)
Supplement: Supplementary file 1 [file jop-166-1974-s001.pdf]

Supplementary materials for *Interpersonal dyadic influences on transitions between pain states: a narrative review and synthesis*.

Table of Contents

Search strategy 2

GRADE table 4

Search strategy

Embase <1974 to 2022 July 07>

1 exp Pain/ 1515733
2 exp Fibromyalgia/ 23289
3 exp *Headache/ or exp Headache Disorders/ or exp Migraine Disorders/ 347249
4 (neuralgia or neuropath* or arthriti*).ti,ab,kw. 522757
5 1 or 2 or 3 or 4 1928489
6 (Cohort* or trial or RCT or random* or prospective or prognostic or diary or experiment* or longitudinal or ecological momentary assessment).ti,ab. 6884106
7 cohort analysis/ 860670
8 randomized controlled trial/ 716341
9 prospective study/ 776674
10 longitudinal study/ 174560
11 6 or 7 or 8 or 9 or 10 7248160
12 (protocol or cross-section* or qualitative or systematic review or meta-analysis or meta-synthesis or narrative review or case series or case stud*).ti,ab. 2163862
13 cross-sectional study/ 491608
14 qualitative research/ 101839
15 12 or 13 or 14 2290668
16 11 not 15 6470580
17 rodent/ 36897
18 exp animal experiment/ 2867222
19 nonhuman/ 6956613
20 (rodent# or rat# or mouse or mice).ti,ab. 5771792
21 17 or 18 or 19 or 20 10664447
22 16 not 21 4218248
23 (partner# or spouse or husband# or wife or wives or significant other# or couple# or dyad# or married or marriage or mother# or father# or parent* or family or families or parent-child or paternal or maternal or child* or adolescen* or p?ediatric or teen or youth or young person# or young adult# or juvenile or infant or toddler# or baby or babies or neonatal or sibling# or brother# or sister#).ti,ab. 4983644
24 family attitude/ or family conflict/ or family coping/ or family functioning/ or family interaction/ 14144
25 23 or 24 4985091
26 (communicat* or social support* or support* or relationship or protect* or behavio?r or respon* or non-verbal or verbal or body language or congruen* or attitude or empath* or listen* or belie* or respect* or compassion* or care or caring or reassur* or validation or invalidation or solicitous*).ti,ab. 14244490
27 interpersonal communication/ 171677
28 nonverbal communication/ 9117
29 26 or 27 or 28 14298187
30 (attachment or enmeshment or reinforc* or contingen* or operant or learn* or punish* or reward or emotion* or comfort* or hold* or sooth* or guilt or confirmation).ti,ab. 1634120
31 (Sex* or romantic* or intima* or collusion or neglect or abus* or adverse childhood e* or trauma*).ti,ab. 1890307
32 29 or 30 or 31 15817521
33 5 and 22 and 25 and 32 26001
34 (vaccin* or virus or viral or infect* or immunogenicity or covid-19 or covid or sars-2-cov or influenza or plasma or blood or tissue or serum or urin* or biomarker# or intravenous or

intramuscular or inflammatory marker# or biopsy or laparoscopy or colonoscopy).ti,ab,kw. 9861119

35 (dosage# or dose# or milligram# or tolerance or tolerability or topical or safety or side-effects or phase or corticosteroids or fentanyl or pharmacokinetic# or pharmacodynamic# or calcium channel or inhibitor or antagonist or drug profile or il-6 or interleukin or protein or genetic or phenotype# or mutation or polymorphism or polygenic or antibod*).ti,ab,kw. 9561326

36 34 or 35 15690783

37 33 not 36 10861

38 limit 37 to human 10360

GRADE table

Mechanism	Studies *(n)*	Dyads *(n)*	Risk of bias *(n high risk)*	Inconsistency *(concerns)*	Indirectness *(concerns)*	Other	GRADE
Parent anxiety	7	11479	7 | 7	some concerns	some concerns	None	Moderate
Parent depression	11	12668	9 | 11	serious concerns	some concerns	None	Low
Parent distress	2	534	2 | 2	some concerns	serious concerns	None	Very low
Parent stress	1	2230	1 | 1	NA	NA	None	Very low
Parent personal strain	1	204	1 | 1	NA	NA	None	Very low
Parent hope	1	50	1 | 1	NA	NA	None	Very low
Parent psychosocial functioning	1	670	1 | 1	NA	NA	None	Very low
Parent catastrophising	13	1811	8 | 13	serious concerns	serious concerns	None	Very low
Parent intolerance of uncertainty	2	308	1 | 2	no concerns	no concerns	None	Very low
Parent cognitive reappraisal	1	56	1 | 1	n/a	n/a	None	Very low
Parent protective behaviours	10	1362	3 | 10	some concerns	no concerns	None	Low

**Mechanism**	**Studies** *(n)*	**Dyads** *(n)*	**Risk of bias** *(n high risk)*	**Inconsistency** *(concerns)*	**Indirectness** *(concerns)*	**Other**	**GRADE**
Parent minimising behaviours	3	396	0 | 3	some concerns	no concerns	None	Very low
Parent monitoring behaviours	3	396	0 | 3	no concerns	some concerns	None	Low
Parent distracting behaviours	3	396	0 | 3	no concerns	some concerns	None	Low
Parent solicitous behaviours	2	330	0 | 2	some concerns	some concerns	None	Very low
Parent pain behaviours	2	416	1 | 2	no concerns	no concerns	None	Very low
Partner solicitous behaviours	4	511	1 | 4	some concerns	some concerns	None	Very low
Partner punishing behaviours	4	562	2 | 4	some concerns	some concerns	None	Very low
Partner distracting behaviours	2	330	1 | 2	no concerns	no concerns	None	Very low
Partner empathic behaviours	2	181	0 | 2	some concerns	serious concerns	None	Very low
Partner support	3	3781	1 | 3	some concerns	serious concerns	None	Very low
Partner confidence in significant other	3	393	0 | 3	some concerns	no concerns	None	Very low
Partner motivation to help	1	70	1 | 1	n/a	n/a	None	Very low

**Mechanism**	**Studies** *(n)*	**Dyads** *(n)*	**Risk of bias** *(n high risk)*	**Inconsistency** *(concerns)*	**Indirectness** *(concerns)*	**Other**	**GRADE**
Partner validation	1	20	0 | 1	n/a	n/a	None	Very low
Goal conflict	1	68	0 | 1	n/a	n/a	None	Very low
Partner depressive symptoms	1	133	1 | 1	n/a	n/a	None	Very low
